# Supplementary material for: Generation of Primordial Germ Cell-like Cells from iPSCs Derived from Turner Syndrome Patients
Source: Cells. 2021 Nov 10;10(11):3099. doi: 10.3390/cells10113099 (PMC8624672; doi:10.3390/cells10113099)
Supplement: Supplementary file 1 [file cells-10-03099-s001.zip › cells-1437958-supplementary.pdf]

Article

Generation of primordial germ cell-like cells from iPSCs derived from Turner syndrome patients

Aline Fernanda de Souza[1,2*]; Fabiana Fernandes Bressan[1]; Naira Caroline Godoy Pieri[1,]; Ramon Cesar Botigelli[1,3]; Tamas Revay[4]; Simone Kashima Haddadd[5]; Dimas Tadeu Covas[5], Ester Silveira Ramos[6]; Willian Allan King[2], Flavio Vieira Meirelles[1]

Supplementary Table, Figures and Legends

Table S1. Efficiency of expansion of PBMCs.

Days of PBMCs expansion	Number of the Cells Live D0	Number of the Cells Live D3	Number of the Cells Live D6	Number of the Cells Live D9	Number of the Cells Live D10
**Male Control**	9×10^6	3.6×10^6	1.17×10^6	1.41×10^6	1.64×10^6
**Female Control**	3.2×10^7	9.6×10^5	1.8×10^6	2.7×10^6	4.2×10^6
**Pt.1**	3.2×10^6	1.82×10^6	1.3×10^6	1.99×10^6	4.1×10^6
**Pt.2**	8×10^6	5.2×10^6	3.53×10^6	5.1×10^6	3.8×10^6
**Pt.3**	4.4×10^6	3.1×10^6	2.61×10^6	3.42×10^6	4.6×10^6
**Pt.4**	3.4×10^6	2.18×10^6	4×10^5	1.34×10^6	1.53×10^6
**Pt.5**	3.9×10^6	1.17×10^6	6×10^5	9.9×10^5	1.2×10^6

PBMCs= peripheral mononuclear blood cells; Pt= Patient; D=day.

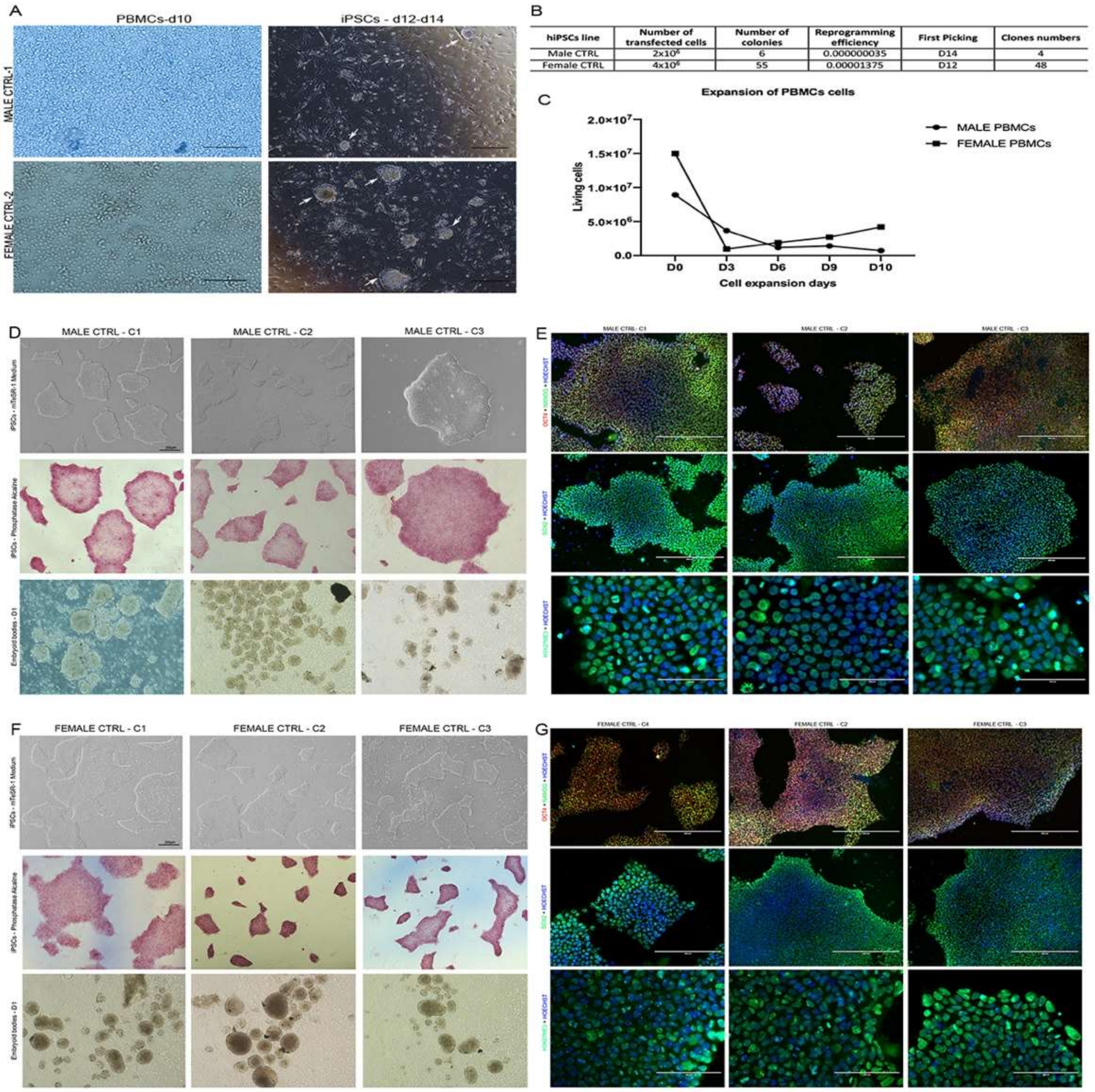

hiPSCs line	Number of transfected cells	Number of colonies	Reprogramming efficiency	First Picking	Clones numbers
Male CTRL	$2x10^6$	6	0.000000035	D14	4
Female CTRL	$4x10^6$	55	0.00001375	D12	48

Supplementary Figure S1. Represent the process of reprogramming of PBMCs- CTRLs into hiPSCs and characterization of hiPSCs male and female controls. A. PBMCs with ten days of expansion in a specific medium for enrichment erythroblastic populations; the emergence of the first hiPSCs colonies maintained in hESCs medium on MEF, showed after 12 days. B. Table of efficiency of reprogramming of PBMCs by episomal vectors. (C) Graphic of representative cell growth data showing number of the live cells during expansion in a culture for nonlymphoid cell enrichment. The data was compiled from at least three independent experiments. D, E, F and G.Characterization of hiPSCs controls. D and F. Phase contrast image showed hiPSCs maintained in mTeSR-1 medium on Geltrex; positively stain of alkaline phosphatase; embryoid body formation after 24 hours of differentiation. Scale bars 200µm.E and G. Immunofluorescence analyzed showing the expression of pluripotency markers OCT4, SOX2, NANOG in hiPSCs controls (p20) and ESCs-H1 (p30). Nuclei were stained with Hoechst (blue). Scale bars: 200µm and 100µm. The data was compiled from at least three technical replicates.

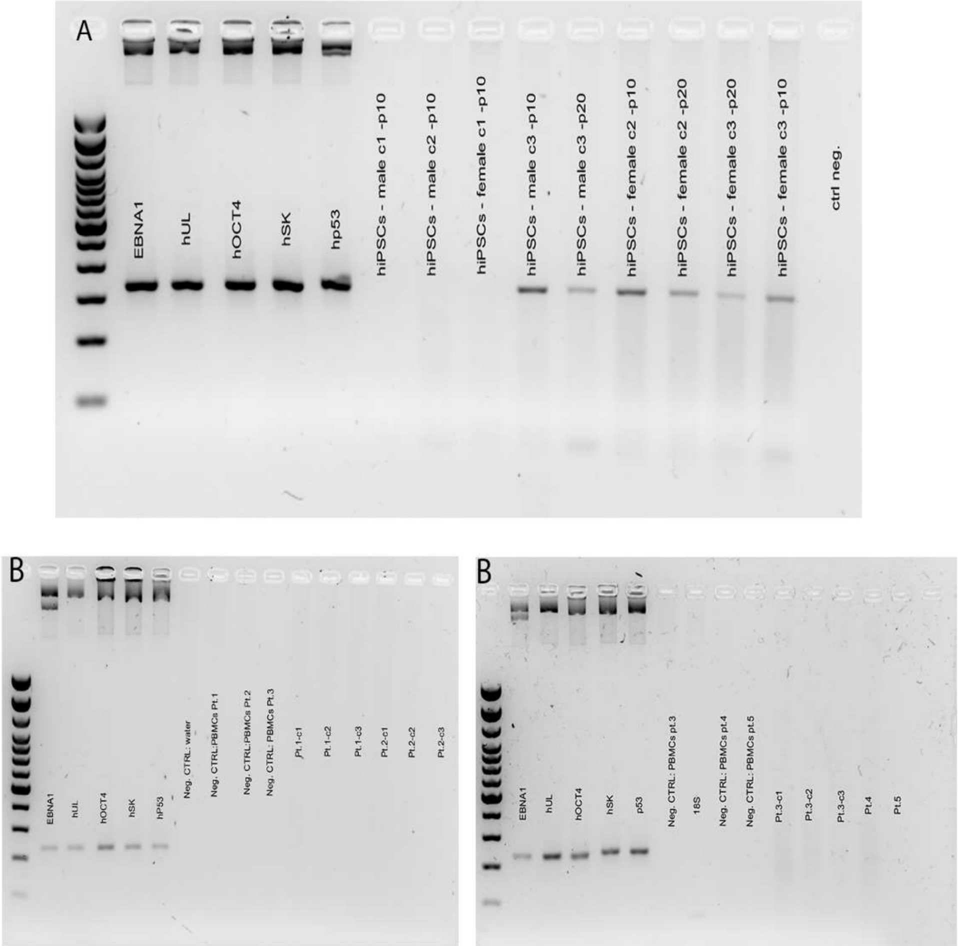

Supplementary Figure S2. Screening for occurrences of vector spontaneous integrations. A. Image of 1.5% agarose gel stained with ethidium bromide representative of the electrophoresis of the episomal vectors amplification reaction in hiPSC controls lines and colonies generated showing free integration of the vectors at hiPSCs male control colonies C1 and C2, and hiPSCs female control colony C1. hiPSCs male control colony C3 and hiPSCs female control, colonies C2 and C3 showed the presence of epissomal vectors at p10 and p20. B. Image of 1.5% agarose gel stained with ethidium bromide representative of the electrophoresis of the episomal vectors amplification reaction in hiPSCs-TS lines and colonies generated showing free integration of the vectors. Data was compiled from at least three technical replicates.

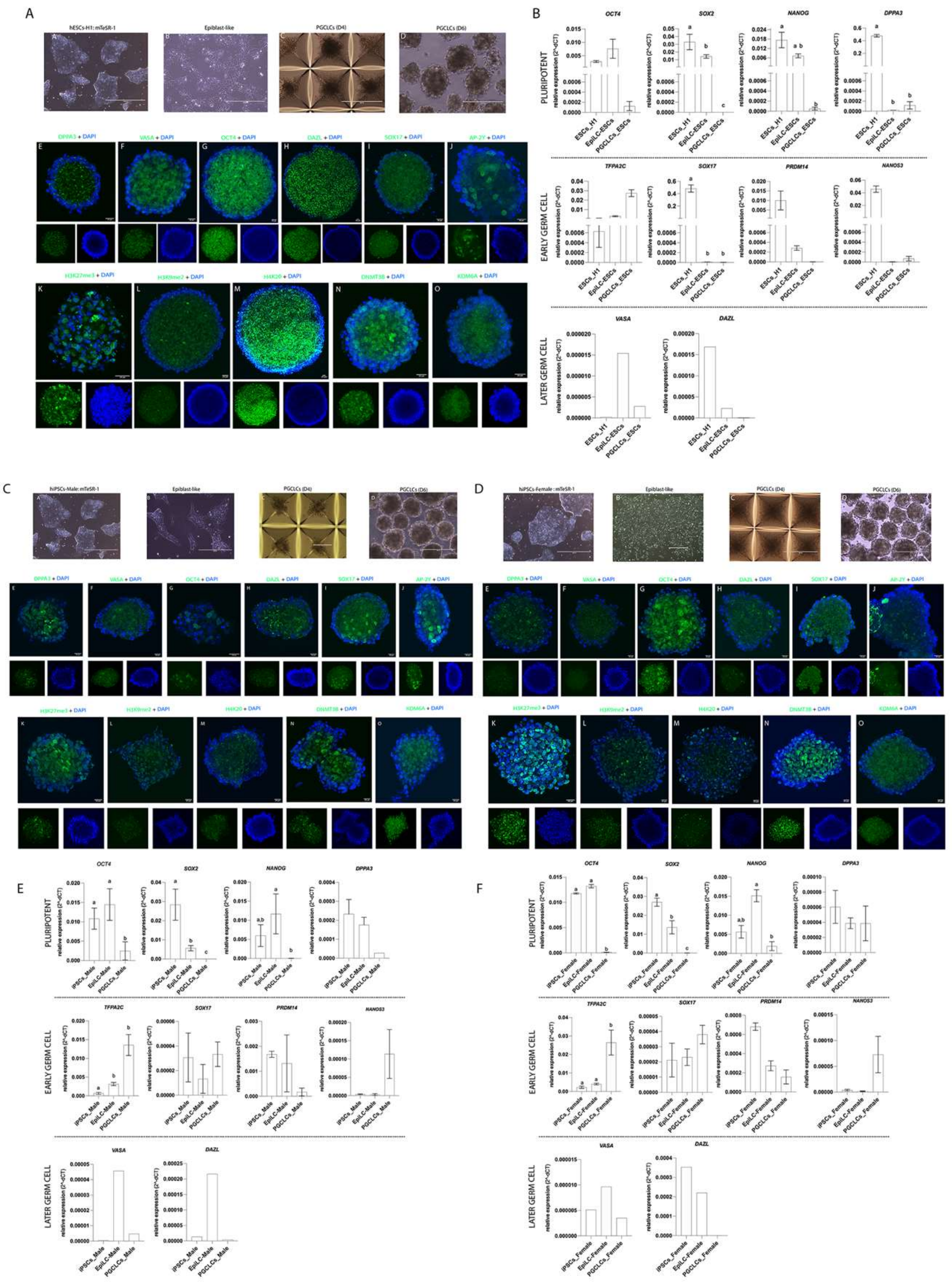

Supplementary Figure S3. Dynamics of pluripotent and germ cells markers during PGCLCs controls induction. A, C and D. ESCs-H1 (p30), hiPSCs male and female controls (p20) showed phase contrast image cells maintained in mTeSR-1 medium on geltrex; (b) ESCs-H1, hiPSCs male and female controls stimulated via hEpiLCs; (c) PGCLCs controls (D4) cultured in agree

well plated. Scale bars: 200µm; (d) PGCLCs controls (D6) isolated for further characterization. Scale bars: 400µm. (e, f, g, h, I and j) Immunofluorescence analyzed showing the expression of pluripotency and germ cells markers DDPA3, VASA, OCT4, DAZL, SOX17 AND AP-2γ. Nuclei were stained with Hoechst (blue). Scale bars s: 20µm. (k, l, m, n and o) Immunofluorescence analyzed showing the epigenetic profile of histones H3K27me3, H3K9me2 and H4K20; and DNMT3B and KDM6A protein markers. Nuclei were stained with Hoechst (blue). Scale bars: 20µm. The data was compiled from at least two technical replicates for all stanning. B, E and F. ESCs-H1 (p30), hiPSCs male and female controls (p20), hEpiLCs controls (D2), and PGCLCs controls (D6) quantification of the relative expression of *OCT4, SOX2, NANOG, DPPA3, TFAP2C, SOX17, PRDM14, NANOS3, VASA* and *DAZL* genes associated with germ cells development. Individual reactions of qRT-PCR were normalized to the *B-ACTIN* gene. The data was compiled from at least three technical replicates for ESCs, hiPSCs, hEpiLCs and PGCLCs. P values were calculated by Two-way ANOVA, followed by Tukey's multiple comparisons test as appropriate. (letters a-b-c show difference between groups). Erros bars denote represent SEM.

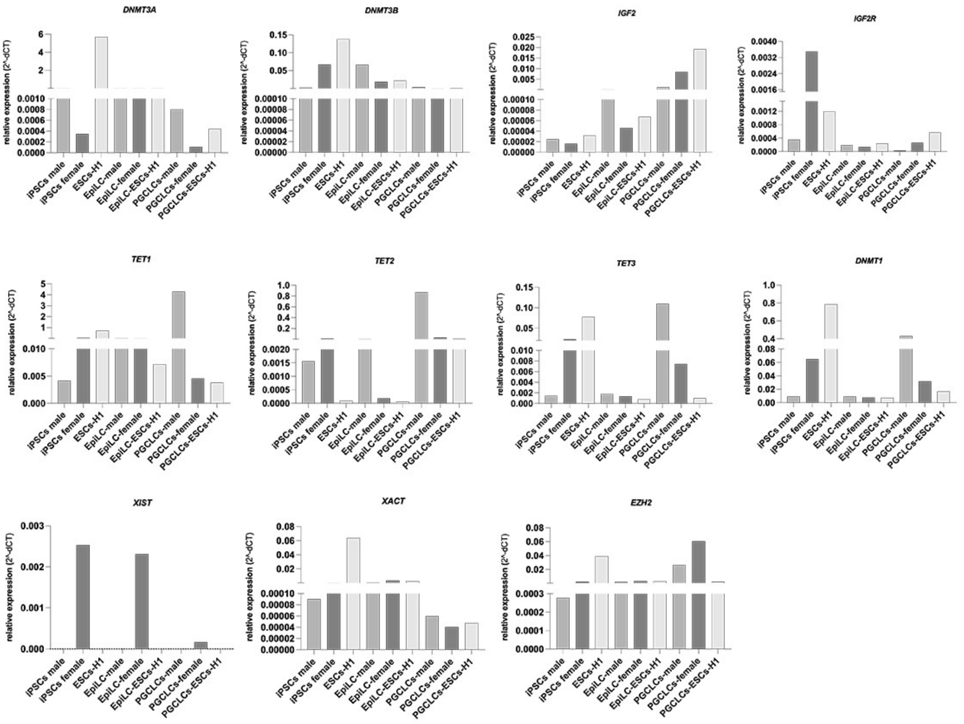

Supplementary Figure.S4: Epigenetic profile during PGCLCs controls induction. hiPSCs male and female controls (p20), ESCs-H1 (p30), hEpiLCs controls (D.2), and hPGCLCs controls (D.6) quantification of the relative expression of genes associated with epigenetic marks. Individual reactions of qRT-PCR were normalized to the *B-ACTIN* gene. The data was compiled from at least three technical replicates. Results are represent individually, and no statistical values were calculated.
